# Single-Point but Not Tonic Cuff Pressure Pain Sensitivity Is Associated with Level of Physical Fitness – A Study of Non-Athletic Healthy Subjects

**DOI:** 10.1371/journal.pone.0125432

**Published:** 2015-05-01

**Authors:** Dag Lemming, Björn Börsbo, Anna Sjörs, Eva-Britt Lind, Lars Arendt-Nielsen, Thomas Graven-Nielsen, Björn Gerdle

**Affiliations:** 1 Department of Pain and Rehabilitation Center and Department of Medical and Health Sciences, Linköping University, SE-581 85 Linköping, Sweden; 2 Center for Sensory-Motor Interaction (SMI), Laboratory for Experimental Pain Research, Department of Health Science and Technology, Aalborg University, 9220 Aalborg, Denmark; University Zurich, SWITZERLAND

## Abstract

Exercise is often used for pain rehabilitation but the link between physical activity level and pain sensitivity is still not fully understood. Pressure pain sensitivity to cuff algometry and conditioned pain modulation (CPM) were evaluated in highly active men (n=22), normally active men (n=26), highly active women (n=27) and normally active women (n=23) based on the Godin Leisure-Time Exercise Questionnaire. Cuff pressure pain sensitivity was assessed at the arm and lower leg. The subjects scored the pain intensity on an electronic Visual Analogue Scale (VAS) during ten minutes with 25 kPa constant cuff pressure and two minutes with zero pressure. The maximal VAS score and area under the VAS-curve were extracted. Pressure pain thresholds (PPT) were recorded by manual pressure algometry on the ipsilateral tibialis anterior muscle before, during and after the tonic arm stimulation. Tonic cuff stimulation of the arm and leg resulted in higher VAS peak scores in women compared with men (p<0.04). In all groups the PPTs were reduced during and after the cuff stimulation compared with baseline (p=0.001). PPT were higher in men compared with women (p=0.03) and higher in highly physical active compared with normal active (p=0.048). Besides the well-known gender difference in pressure pain sensitivity this study demonstrates that a high physical fitness degree in non-athletic subjects is associated with increased pressure pain thresholds but does not affect cuff pressure pain sensitivity in healthy people.

## Introduction

Regular physical training is believed to have beneficial effects on general health and cardiovascular risk, there is also sufficient evidence to recommend physical training as a treatment modality for patients with chronic musculoskeletal pain [1,2]. Reduced pain sensitivity during and after different types of standardized physical exercises have been reported [3,4] where exercise-induced hypoalgesia is most pronounced in strenuous physical activity [5]. The underlying mechanisms of how physical activity modulates pain perceptions are not fully understood. Possible explanations are via stimulation of the endogenous opioid system or stimulation of baroreceptors by increased blood pressure resulting in increased supraspinal inhibition [6]. Furthermore it has been suggested that psychological factors are involved in the relationship between physical activity and pain perception [7]. Reduced pain responses to the cold-pressor task were found in individuals who perform more strenuous physical activity and that this was correlated with a lower degree of catastrophizing [7]. Athletes seem to develop long-term alterations in pain perception mainly demonstrated as increased tolerance to mechanical stimuli, whereas pain thresholds show inconsistent changes in comparison to normally active control subjects [8]. Furthermore, there are recent results suggesting that the endogenous pain inhibitory system may be less responsive in athletes during conditioned pain stimulation using tonic heat as test stimuli and the cold-pressor task as conditioning stimuli [9] potentially due to an already active descending inhibitory control. However, to our best knowledge it is not known if a higher fitness level in healthy non-athletes is associated with less pain sensitivity than in healthy subjects with lower fitness level.

Females are known to demonstrate higher sensitivity to some pain modalities as compared with men both in clinical and experimental studies [10,11] and this difference probably develops during puberty [12]. However, experimental studies do not show a consistent pattern concerning sex differences. Recently Racine et al. [13] concluded that laboratory research has not been successful in producing a clear and consistent pattern of sex differences in the pain system responses [13]. Several studies have investigated the relationship between sex and pressure pain sensitivity and most results find that females have significantly lower pain thresholds than males [14,15]. One possible mechanism contributing to sex differences in pain sensitivity might be differences in activation of conditioned pain modulation (CPM); the decreased sensitivity response to a conditioning pain stimulus (e.g., pressure, cold water or ischemia) [16]. Larger CPM effect has been found in males compared to females; however, the sex differences in CPM effect may depend on both the methodology used in the experiment and the modes of measurement of the effect [16]. In conclusion, although most studies indicate that women have higher pain sensitivity the literature is not in consensus and more studies are needed.

Computerized cuff pressure algometry is a tool for assessment of pressure-pain sensitivity and mechanisms related to central modulation of pain (i.e., temporal and spatial summation of pressure-pain) [17]. Cuff algometry mainly assesses sensitivity in deep somatic tissue and is less biased by inter- and intra-examiner variability than conventional handheld pressure algometry technique [18,19]. Previously cuff algometry has demonstrated increased pressure pain sensitivity in fibromyalgia [20], whiplash associated disorder [21], lateral epicondylalgia [22], and chronic pain after revision knee arthroplasty [17].

Based on the above brief review of the literature we hypothesized that being woman and/or having a low fitness level were associated with lower pain thresholds. Thus, the aim of this explorative study was to investigate single-point and cuff pressure-pain sensitivity comparing four groups of healthy non-athletic subjects based on sex and level of physical activity.

## Materials and Methods

### Subjects

The subjects were recruited through advertisement in the local newspaper. Both normally trained and well-trained subjects were recruited. Inclusion criteria were age between 20 and 65 years, and pain-free. A brief medical history was taken that included any current or previous presence of pain. Power analysis for this study suggested a sample size of 50 individuals in each group when looking for gender differences. We hypothesized a similar sample size would be sufficient for detecting differences related to physical activity level. (Power 0.8 and two-tailed significance level p<0.05). For pairwise comparisons the power analysis based on data concerning pressure pain thresholds (PPT; assuming a difference of 50 kPa and a standard deviation of 100 kPa) suggested 33 subjects (Power 0.8 and two-tailed significance level p<0.05).

### Ethics statement

The study was conducted in accordance with the Declaration of Helsinki. The study was granted ethical clearance by the Linköping University Ethics Committee (2011/102-31), and all participants gave informed written consent. The approval of the Ethics Committee included the possibility for the subjects of receiving 400 SEK as compensation for the participation in the study.

### Physical activity level

Godin Leisure-Time Exercise Questionnaire (GLTEQ) was used to estimate the physical activity level; it contains four questions where the person states how many times weekly he/she is doing “strenuous”, “moderate” and “mild” exercise respectively. The different intesities are described with exampels in the questionnaire. A total leisure activity score is calculated by the times per week stated for the different intensities muliplied with 9 for strenouos, 5 for moderate and 3 for mild. A high score indicate higher intensity and higher frequency of weekly leisure-time activities [23,24]. Normal physical activity level was defined as GLTEQ scores less or equal than the median split of GLTEQ scores for all subjects. Consequently, subjects with GLTEQ scores higher than the median split of GLTEQ scores were categorised in the high activity group.

### Experimental protocol

The dominant “writing hand” side was chosen for all assessments. Subsequently, tonic cuff stimulation was performed on the dominant arm and later on the dominant leg. Before, during and after the cuff stimulation on the arm, pressure pain thresholds (PPT) were recorded on the ipsilateral tibialis anterior muscle. The time elapsed between the arm and leg cuff stimulation was no less than ten minutes.

### Cuff algometry

The experimental setup consisted of a double chamber 13-cm wide tourniquet cuff (a silicone high-pressure cuff, separated lengthwise into two equal-size chambers, VBM Medizintechnik GmbH, Sulz, Germany), a computer-controlled air compressor, and an electronic visual analogue scale (Nocitech, Denmark). The cuff was connected to the compressor and wrapped around the heads of biceps and triceps muscles of the arm or around the mid-portion of the triceps surae muscles of the leg. The maximum pressure limit used was 25 kPa in both regions and maintained for 10 min. Cuff inflation to 25 kPa pressure was instantaneous, as well as the deflation after 10 min. The stimulation could be aborted at any time by the subject using a push button or the experimenter via the computer. During the cuff stimulation the subjects scored the pain intensity simultaneously on an electronic visual analogue scale (VAS) which was sampled at 10 Hz. Zero and 10 cm extremes on the VAS were defined as “no pain” and as “worst possible pain”, respectively.

The VAS was recorded for 12 min (i.e. including 2 min recording with zero pressure) The maximum pain intensity (VAS-peak) and time to VAS-peak were extracted. If a subject aborted the 12 minute assessment prematurely, the time elapsed (sec) was registered (abort-time). Individual slopes in pain intensity raise and fall were calculated from the start of inflation to the end of inflation (Slope-tonic), and from the end of inflation to the end of the assessment (Slope-tail). Areas under the VAS-curve were calculated based on raw data. These areas were; for the entire assessment (AUC-all), for the 10 min with cuff inflation (AUC-tonic) and for the 2 min with zero pressure (AUC-tail). In addition, to investigate the increase in pain during tonic stimulation relative to the initial pain intensity, the area under the VAS-curve during tonic stimulation using the mean pain intensity the first 30 sec of cuff inflation as ground level was calculated (AUC-tonic norm).

### Handheld Pressure algometry

Pressure pain thresholds were determined using a manual pressure algometer (Somedic AB, Sweden) mounted with a probe (with a contact area of 1 cm^2^) on the muscle belly of the ipsilateral tibialis anterior muscle. The pressure was increased by 30 kPa/s until the subject perceived pain and pushed a stop-button. The PPT was defined as the mean of two trials obtained with minimum 30 sec interval. The PPT was measured at baseline during the initial part of the experiment, immediately before the tonic stimulation (PPT-0), after two minutes of tonic cuff stimulation (PPT-2) and 15 minutes (PPT-15) after beginning tonic stimulation of the arm (i.e. three minutes after ending the continuous VAS recording).

### Statistics

Statistical analyses were made using IBM SPSS (version 20.0; IBM Corporation, Route 100 Somers, New York, USA). P≤0.05 was used as level of significance in all analyses. Data in text and tables are presented as mean values ± standard error of the mean (SEM).

*Paired t-test* was used for the comparisons of the variables of the tonic test between arm and leg. *T-test* was used for comparisons of variables between independent factors (i.e. sex and physical activity level). A *two-way analysis of variance (ANOVA)* was used for investigating main and interaction effects of sex and physical activity level on cuff pressure variables.

*ANOVA* with *Bonferroni post-hoc test* was used for comparisons between the four groups. *Factorial repeated measures ANOVA* (Split-Plot or Mixed Between-Within Subjects) was used when testing PPTs over time (PPT0-PPT15) and investigating the main effects of sex and fitness level.

## Results

### Subjects

For the whole sample the measures of central tendency for GLTEC were; mean = 47.8±2.6 and median = 45.5(6–145). Based on a median split, normal physical activity level was defined as GLTEQ score≤45 and high physical activity level as GLTEQ score>45.

Four groups were defined based on sex and physical activity level; highly active men (HAM; n = 22), normally active men (NAM; n = 26), highly active women (HAW; n = 27) and normally active women (NAW; n = 23).

There was no significant difference in mean age between the four groups (Table 1). For the anthropometric data were found significant sex differences between the four groups. Weight and BMI also differed with respect to fitness level. No differences existed in systolic and diastolic blood pressures. As intended, significant differences were found across groups for exercise time/week (Table 1).

**Table 1:** Mean values (±SEM) of back-ground data in the four groups; highly active men (HAM), normally active men (NAM), highly active women (HAW) and normally active women (NAW).

Groups	HAM	NAM	HAW	NAW	ANOVA	TWO-WAY ANOVA			
Variables	(n = 22)	(n = 26)	(n = 27)	(n = 23)		Sex	Fitness		Interaction
Age	30.6±1.9	36±2.4	34.8±1,8	35.7±2.5	ns	0.388		0.152	0.308
Height (cm)	181±1	182.2±1.5	166.3±1.3	169.6±1.2	<0.001*	<0.001*		0.094	0.439
Weight (Kg)	79.6±1.9	82.9±1.6	62±1.6	68.5±1.6	<0.001*	<0.001*		0.005*	0.352
BMI	24.3±2.7	25.0±1.9	22.3±2.1	24.0±3.0	0.001*	0.004*		0.017*	0.273
Systolic BP (mm Hg)	136.9±3.2	131.6±1.9	125.4±6.2	123.3±1.8	ns	0.014*		0.352	0.684
Diastolic BP (mm Hg)	77.9±1.5	78.2±0.9	79.1±2.3	75±1.5	ns	0.539		0.253	0.179
GLTEQ	71.2±4.9	26.3±2.4	65.8±3.5	28.7±1.9	<0.001*	0.656		<0.001*	0.248

Two types of statistical analyses were made. First an ANOVA to compare the four groups with respect to variables displayed and Secondly the result of the two way ANOVA displaying the effects (p-values) of sex, fitness level and interaction (sex*fitness level) for the variables.

* denotes significant group difference or effect.

Body Mass Index (BMI), Blood pressure (BP), Godin Leisure-Time Exercise Questionnaire (GLTEQ), Exercise times/week (GLTEQ4).

Few of the subjects were smokers or moist snuffers and no group differences existed between groups with respect to proportions of this item.

### Cuff algometry

**Fig 1:**
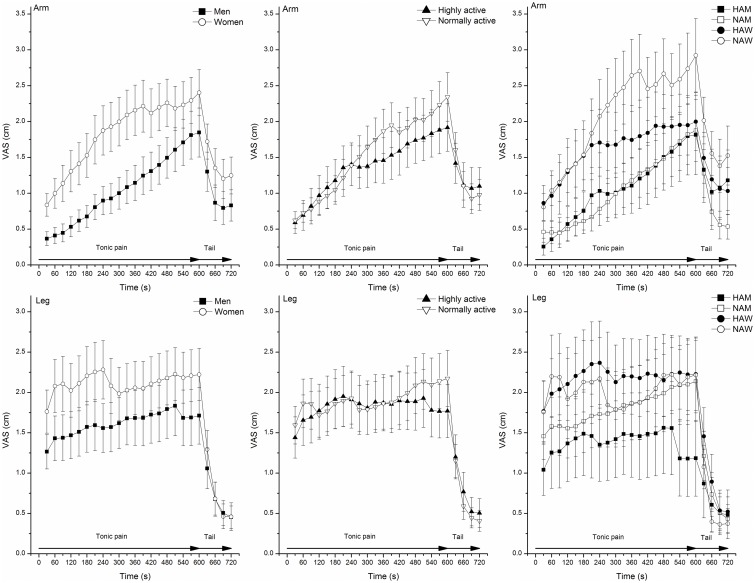
Mean pain intensity (±SEM) every 30 s during and after tonic pressure pain in highly active men (HAM), normally active men (NAM), highly active women (HAW) and normally active women (NAW).

Pain intensities during and after tonic pain stimulation are presented in Fig 1.

### VAS-peak

No difference in VAS-peak-arm and VAS-peak-leg was found. The two-way ANOVA of VAS-peak-arm showed a significant main effect for sex (p = 0.013) but no significant effects of fitness level or interaction (Table 2). One-way ANOVA revealed a difference between normally active men and women (p = 0,043). Similar results were found for VAS-peak-leg; sex (p = 0.038), however no significant effects of fitness level, interaction or differences between the four groups (Table 2).

**Table 2:** Variables (Mean values (±SEM)) of the tonic tests of the arm and the leg in the four groups; highly active men (HAM), normally active men (NAM), highly active women (HAW) and normally active women (NAW).

	Group										
	HAM		NAM		HAW		NAW		TWO-WAY ANOVA		
	Mean	SEM	Mean	SEM	Mean	SEM	Mean	SEM	Sex	Fitness	Interaction
Variables									(p-value)	(p-value)	(p-value)
***Arm***											
VAS-peak	2.43	.59	2.20	.44	3.07	.58	4.35	.58	0.013*	0.340	0.177
Time to VAS-peak	547	40	505	35	382	46	529	40	0.088	0.196	0.023
AUC-tonic	644	208	636	160	983	255	1067	175	0.734	0.839	0.126
AUC-tail	138	52	93	27	143	41	194	35	0.183	0.939	0.231
AUC-all	782	254	729	183	1126	274	1261	196	0.065	0.861	0.690
AUC-tonic norm	491	163	360	95	465	174	581	169	0.525	0.963	0.422
Slope-tonic	0.16	0.05	0.16	0.04	0.11	0.03	0.23	0.05	0.860	0.113	0.164
Slope-tail	-0.09	0.15	-0.49	0.14	-0.31	0.19	-0.36	0.32	0.829	0.279	0.386
***Leg***											
VAS-peak	2.44	.68	2.69	.46	3.47	.52	4.06	.64	0.038*	0.470	0.769
Time to VAS-peak	320	54	219	50	299	47	332	49	0.357	0.503	0.183
AUC-tonic	717	246	1086	250	1307	272	1129	242	0.226	0.715	0.295
AUC-tail	74	36	87	27	102	33	66	15	0.892	0.599	0.423
AUC-all	791	276	1172	267	1409	289	1195	254	0.253	0.765	0.288
AUC-tonic norm	147	206	212	139	247	139	278	170	0.612	0.768	0.919
Slope-tonic	0.01	0.07	0.07	0.03	0.02	0.03	0.07	0.05	0.838	0.203	0.904
Slope-tail	-0.26	0.17	-0.54	0.15	-0.64	0.20	-0.45	0.21	0.438	0.807	0.225

* denotes significant effect.

Area under curve for the; 10 minutes of cuff inflation, 2 minutes with zero cuff pressure, total area, normalized area during cuff inflation (AUC-tonic, AUC-tail, AUC-all, AUC-tonic norm). Slope from the start to the end of inflation (Slope-tonic), Slope from the end of inflation to the end of the assessment (Slope-tail).

### Time to VAS-peak

A significant difference in time to VAS-peak was found between arm and leg (Arm: 486±21 vs. Leg: 290±25; p<0.001); the time was shorter in the leg than in the arm. The two variables intercorrelated significantly (r = 0.282; p = 0.005). The analyses of time to VAS-peak showed no significant main effects for sex, fitness level or interaction neither in the arm nor in the leg (Table 2).

### Areas under curve (AUC-all/-tonic/-tonicnorm and -tail)

For the AUC variables shown in Table 2 existed significant differences between arm and leg. The *AUC-all* (both tonic stimulation and the tail after stimulation) was significantly larger in the leg than in the arm: Arm: 917±113 vs. Leg: 1160±138; p = 0.015. The two variables intercorrelated significantly (r = 0.714; p<0.001). The *AUC-tonic* was significantly larger in the leg (Arm: 787±101 vs. Leg: 1076 ±119; p = 0.003). The two variables intercorrelated significantly (r = 0.693; p<0.001). The reverse relationship was found for the *AUC-tonic norm* (Arm: 434±73 vs. Leg: 222 ±79; p = 0.006). The two variables intercorrelated significantly (r = 0.506; p<0.001). For the *AUC- tail* was found a significantly larger area in the arm (Arm: 131±19 vs. Leg: 83 ±15; p = 0.004). The two variables intercorrelated significantly (r = 0.600; p<0.001). The analyses of the areas under curve variables including the normalized area showed no significant main effects for sex, fitness level or interaction neither in the arm nor in the leg (Table 2).

### Slope during and after tonic stimulation

The Slope-tonic was steeper in the arm than in the leg (Arm: 0.16±0.02 vs. Leg: 0.04±0.02; p<0.001). The two variables intercorrelated significantly (r = 0.598; p<0.001). The Slope-tail did not differ between arm and leg. The analyses of the slopes showed no significant main effects for sex, fitness level or interaction neither in the arm nor in the leg (Table 2).

### Abort-time

Two subjects aborted prematurely during tonic stimulation of the arm (elapsed time: 378 and 497 sec; both normally active women) and 4 subjects during tonic stimulation of the leg (elapsed time: 9, 101, 243 and 541 sec; three normally active women and one highly active man (541 sec)). The two women aborting their arm-recordings also aborted corresponding leg-recordings (9 and 101 sec), thus four subjects in total aborted any session.

### PPT assessment

At PPT-baseline male subjects had significantly higher thresholds than female subjects (590±21 vs. 523±19; p = 0.019), at PPT-0 highly active subjects had significantly higher thresholds than normally active subjects (586±19 vs. 531±20; p = 0.049). PPT was not significantly different between the four groups at PPT-baseline, PPT-0 or PPT-2. At PPT-15 there was a difference between highly active men and normally active women (p = 0.017). Using factorial repeated measures ANOVA were found that PPT (Table 3) decreased over time (p = 0.001) and significant main effects existed for sex (p = 0.030) and fitness level (p = 0.048) but without significant interactions.

**Table 3:** Mean values (±SEM) of pressure pain thresholds (PPT) at the four time points.

	PPT-baseline				PPT-0				PPT-2				PPT-15			
	Sex				Sex				Sex				Sex			
	Men		Women		Men		Women		Men		Women		Men		Women	
	High or normal physical activity		High or normal physical activity		High or normal physical activity		High or normal physical activity		High or normal physical activity		High or normal physical activity		High or normal physical activity		High or normal physical activity	
	HA	NA	HA	NA	HA	NA	HA	NA	HA	NA	HA	NA	HA	NA	HA	NA
Mean	609	576	529	515	618	550	562	509	600	526	536	489	601	516	519	468
±SEM	32	27	26	29	26	29	26	27	30	31	27	29	29	30	26	30

Highly active (HA), Normally active (NA).

## Discussion

Physical activity level described as normal or high did not affect pain sensitivity to tonic cuff pressure in this sample of healthy people in contrast to the pain sensitivity assessed with single point pressure algometry. Sex was not associated with the temporal aspects of tonic pressure sensitivity, but with the maximum pain intensity.

### Sensitivity to tonic pressure and PPT is associated with sex

Most studies have used handheld pressure algometry [25,26] which gives a very short and localised stimulus with minimal equivalent in a physiological context. The cuff algometry stimulates a large muscle volume and induces a sore feeling similar to pain after heavy exercise and as such mimic more muscle ache and pain.

This study showed increased sensitivity to cuff pressure in the arm and in the leg, in terms of increased maximum pain intensity for women compared with men. The same association is evident for pressure pain thresholds. However analysis of the temporal aspects of pain sensitivity did not show any significant differences related to sex. One reason for this somewhat limited finding could be the relatively small sample of healthy people. The sample size calculations prior to the study indicated sufficient number of subjects both for un-paired and paired comparisons but the higher variation of the individual data concerning these measures than assumed may have resulted in reduced power to detect differences. Another explanation could be related to using a low stimulation intensity not adjusted for the individual pain sensitivity (i.e., adjusted with respect to pressure pain detection- and tolerance thresholds). A meta-analysis by Riley et al. [15] concluded the lack of sex differences observed in many studies could be attributed to insufficient statistical power and they recommended that 41 subjects per group were necessary to provide adequate power to measure sex differences. In this respect this study conformed well to the recommended group size. Another reason for the inconsistencies between studies may be related to some gender differences being pain modality specific e.g. gender difference to pressure pain but no difference to thermal pain [10,11,27]. Thus tonic cuff pressure sensitivity may not show the same pattern as sensitivity to single point pressure, even if the experimental modality is pressure, administered in different ways.

### Sensitivity to PPT is associated with physical fitness

Physical fitness was not a variable of importance influencing any of the investigated variables of tonic pain sensitivity. However PPTs were significantly reduced (i.e., increased sensitivity) during repeated measurements in the leg and there was a main protective effect of high physical activity level (i.e., less reduction of thresholds). If physical fitness induces increased tissue hardness, such phenomenon may indeed change the physical tissue properties enough to cause a change in PPTs. A recent study by Finocchietti et al. discussed the importance of the probe related to tissue tension when measuring PPTs [28]. The VAS rating during tonic stimulation was fairly low which may represent a “subthreshold stimulation” for detecting any modulating effects of physical fitness. Earlier studies showing a relationship between physical fitness and pain sensitivity have mostly been performed in an experimental setting measuring pain sensitivity directly after exercise [3,4]. In the present study the level of fitness was based on self-reported exercise level.

The only other study using GLTQ assessing physical activity level, is a study of Goodin et al [7], they found reduced pain at cold-pressor task in persons with a greater amount of strenuous physical activity per week.

### No net effect of conditioned pain modulation

The lack of physical activity level on CPM effect could be explained by the relative low pain intensity of the conditioning stimuli (i.e., 25 kPa or 190 mmHg). In an earlier study by Lemming et al. the same stimulation intensity was used with sufficient differences between patients and controls regarding cuff pain sensitivity [21]. In this study the primary aim was not to evaluate CPM, rather to investigate if 25 kPa would activate CPM. The mean VAS peak recordings for the arm were quite low in all groups together (<3) and this may explain the lack of CPM. A recent study showed a correlation between CPM and VAS peak pain values using three different conditioning stimuli [29]. Repeated single-point measurements may also have induced peripheral sensitization, opposing CPM. Thus, since CPM was not efficiently induced the present data cannot be used to infer or reject a relation between CPM and activity levels.

### Anatomical differences

Sensitivity to tonic pressure was significantly higher in the leg compared to the arm. This finding is consistent with earlier results from subjects with chronic whiplash associated disorder (WAD) and healthy controls where pain tolerance thresholds for pressure were significantly elevated for both groups [21] in the arm compared to the leg.

Tissue volume compressed and/or the density of nociceptors may also be factors affecting sensitivity differently in arm and leg.

### Strengths and limitations

Cuff algometry has not yet been tested in a broader clinical population, but it is expected that the method will prove to be a useful indicator of sensory dysfunction in patients with chronic pain. Cuff algometry offers a flexible tool for assessments and also adds the advantage of studying more dynamic features of pain response. In future studies, pressure stimulation intensities should be related to the individual cuff pressure sensitivity. Using only a questionnaire may not be sensitive enough to reflect the actual level of physical fitness, adding objective measures (e.g., oxygen uptake or accelerometer) would be recommendable in future work.

## Conclusion

This study indicates that physical fitness at a non-athletic level does not affect pain sensitivity to tonic cuff pressure in a sample of healthy people in contrast to the findings of single-point pressure pain sensitivity. Sex is not associated with the temporal aspects of tonic pressure sensitivity.
